# Why epistasis is important for tackling complex human disease genetics

**DOI:** 10.1186/gm561

**Published:** 2014-06-09

**Authors:** Trudy FC Mackay, Jason H Moore

**Affiliations:** 1 Department of Biological Sciences, North Carolina State University, Raleigh, NC 27695, USA; 2 Institute for Quantitative Biomedical Sciences, Geisel School of Medicine, Dartmouth College, Lebanon, NH 03756, USA

## Abstract

**Editorial summary:**

Epistasis has been dismissed by some as having little role in the genetic architecture of complex human disease. The authors argue that this view is the result of a misconception and explain why exploring epistasis is likely to be crucial to understanding and predicting complex disease.

## What is epistasis?

The goal of human genetics is to specify the genotype-phenotype map; that is, to understand how naturally occurring genetic variants jointly act to modulate disease risk. In a typical genome scan (for example, a genome-wide association study), the effect of each variant on the disease trait of interest is interrogated one at a time. The effects of all variants are then summed to deduce the total amount of genetic variation explained by DNA polymorphisms that affect the trait. This additive model of inheritance assumes that the effects of individual variants are independent of the effects of other contributing loci (the genetic background). Epistasis occurs if the effect of one variant affecting a complex trait depends on the genotype of a second variant affecting the trait. For example, consider two loci (A, B), each with two alleles (A_1_, A_2_, B_1_, B_2_). Epistasis would occur, for example, if the A_2_A_2_B_2_B_2_ genotype had a high disease risk, but the eight other possible two-locus genotypes had no effect on risk. This is only one of many possible forms of epistatic interactions between two loci.

## Is there evidence for epistasis for quantitative traits?

Many human diseases and disease-related phenotypes (for example, blood pressure) are quantitative traits. That is, their variation is due to many interacting genetic loci, and the effects of alleles at these loci are highly sensitive to the environmental circumstances to which the individuals are exposed. Quantitative variation in phenotypes and disease risk must result in part from the perturbation of highly dynamic, interconnected and non-linear networks (for example, developmental, neural, transcriptional, metabolic and biochemical networks) by multiple genetic variants [1]; thus, gene-gene interactions are likely. Most evidence for epistatic interactions comes from studies in model organisms. In yeast, nematodes and flies, systematic screens for genetic interactions affecting fitness and quantitative traits have revealed the ubiquity of epistasis [2]. Arguably, though, these interactions could be specific for the large phenotypic effects of mutations and knockdown by RNA interference, not the variants with more subtle effects that segregate in natural populations. However, studies mapping quantitative trait loci (QTLs) in model organisms have often found QTL × QTL interactions, even between QTLs that have no significant effects when these are averaged over all genetic backgrounds. The ability to transfer genomic fragments (entire chromosomes or smaller intervals) between two inbred strains has further revealed pervasive epistasis [3]. Finally, the effects of induced mutations are highly variable in different genetic backgrounds, a phenomenon that can be used to map genes interacting with the focal mutation [2]. If epistatic interactions are so common in ‘simple’ model organisms, it seems unreasonable to assume that they do not occur in humans.

## Why has epistasis been largely ignored in human genetics?

Historically, the genetic analysis of quantitative traits has been purely statistical. The magnitude of variation in a complex trait phenotype can be partitioned into three different types of component: additive components, non-additive components (dominance and epistatic) and environmental variance components [4]. Most quantitative genetic variation is additive, and this has been used to dismiss the relevance of epistasis [5]. However, additive genetic variance can be generated not only by variants with purely additive effects (within and between loci), but also by dominant and recessive variants, and by epistatically interacting variants. This distinction has been articulated as being the difference between biological epistasis (referring to gene action) and statistical epistasis (referring to variance components) [6]. An important factor in understanding this distinction, as explained in more detail below, is that epistatic variance depends on allele frequencies, whereas epistatic gene action does not.

## Why should we consider epistatic interactions in human genetics?

When we are interested in dissecting the genotype-phenotype map for complex traits and common disease, knowledge about epistatic gene action is important. To understand why, consider the case of epistasis illustrated above. If alleles A_2_ and B_2_ are rare, there will be very few diseased individuals in the population and the individual effects of locus A and locus B on disease risk will be very small. In this case, most of the genetic variance will be additive, and the additive effects of these loci will have a negligible impact on the ability to predict the risk of the disease. If there were many other loci for which a similar situation applied, this could generate the appearance of a highly polygenic genetic architecture for the disease, with many segregating alleles with very small effects. This is indeed what has been observed for most complex human diseases and quantitative traits. This phenomenon has been called ‘missing heritability’, because the mapped variants have small effects and together only account for a small fraction of the total genetic variance known to affect the traits. In this scenario, additivity is an emergent property of underlying epistatic interaction networks. Epistasis could thus partially explain the missing heritability. However, knowing the genotypic status of both loci is critical if one happens to be A_2_A_2_B_2_B_2_, and predictive ability when both loci are accounted for would be excellent. In genomic medicine, one does not wish to know the population average effect of a variant at a locus, but rather the effect of one’s particular genotype.

Epistatic gene action will also yield different genetic architectures between populations in which the frequency of the causal alleles vary. For example, consider the same example of epistasis discussed above, but in a population where the frequencies of the A_2_ and B_2_ alleles are high. Such a population would have a high prevalence of disease, and the average effects of both the A and B loci would be appreciable. This would manifest as an unreplicated association in the population in which the A_2_ and B_2_ alleles are rare, yet gene action is identical in the two populations. Thus, the typical requirement that genotype-phenotype associations in humans be replicated across populations will not be met in the presence of epistasis when allele frequencies vary among populations [6].

## What are the challenges for identifying epistatic interactions in human genetics?

The challenges for detecting epistasis in human populations are threefold. The first challenge is statistical. Commonly used parametric statistical methods such as logistic regression have reduced power to detect interactions and often do not converge on accurate parameter estimates. This means that a parametric modeling approach for epistasis requires much larger sample sizes than for tests of the effects of single loci. Methods based on frequentist statistical inference are also less than ideal in the context of epistasis. Frequentist methods include the use of *P* values, which are widely applied to assess the statistical significance of genetic associations. These approaches must balance the increase in type I errors (false positives) that arise due to the astronomical number of tests that must be performed with the increase in type II errors (false negatives), which is associated with the decrease in power that accompanies a stringent significant threshold. Imagine that we genotype 10^6^ SNPs in a population. Even examining only pairwise epistatic interactions would yield approximately 10^12^ statistical tests. An approach known as Bonferroni correction is commonly used to account for multiple testing in genome-wide association studies. However, applying such a correction means that only interactions with extremely large effects could be detected. Another challenge in the analysis of epistasis is computational, and lies in the number of central processing unit cycles that are required to enumerate all possible combinatorial models. In general, it is not possible to test all possible interactions among more than three SNPs at a time in a genome-wide scan. A final challenge is interpretation. High-order interactions with non-additive effects can be difficult to comprehend statistically and perhaps even harder to tie back to biology. Designing combinatorial experiments to validate epistasis models might be more difficult than the analytical challenges.

## Looking forward

The most important short-term goal is to develop, evaluate and employ statistical and computational methods that embrace, rather than ignore, the complexity of the genotype to phenotype map. Parametric statistical approaches have their place in a comprehensive modeling toolbox but their limitations should be recognized. Novel methods such as multifactor dimensionality reduction [7] and machine learning methods such as random forests [8] are capable of modeling non-additive interactions. Combining these novel methods with stochastic search algorithms to explore the combinatorial search space using both high-performance computing and expert knowledge to limit the search space will be required to explore genetic associations in an era of plentiful genome-wide data. Other emerging technologies show great promise for modeling genetic interaction networks. First, artificial intelligence is poised to have a big impact on the genetic analysis of complex traits by generating interesting and unexpected models of genotype to phenotype relationships [9]. Second, visualization can play a critical role in helping the modeler explore, understand and interpret the complexity of the data and the analytical results.

Epistasis is one of several non-mutually exclusive explanations for small effects, missing heritability and lack of replication of top trait-associated variants in different populations in human genome-wide association studies. Determining epistatic gene action in the context of human disease will improve our understanding of the biological systems that underpin variation in disease risk as well as increase the accuracy of individual risk prediction.

## Abbreviations

QTL: Quantitative trait locus; RNAi: RNA interference; SNP: Single nucleotide polymorphism.

## Competing interests

The authors declare that they have no competing interests.

## Acknowledgements

Work in the authors’ laboratories is supported by National Institutes of Health grants R01 GM45146, R01 AA016560, R01 GM076083, R01 GM59469, R01 AG043490 and R21 ES021719 to TFCM, and R01 LM009012, R01 LM010098, R01 AI59694, R01 EY022300, R01 LM011360, P20 GM103534, P20 GM103506 and R42 GM097765 to JHM.
