# Erratum to: Why epistasis is important for tackling complex human disease genetics

**DOI:** 10.1186/s13073-015-0205-8

**Published:** 2015-09-07

**Authors:** Trudy F. C. Mackay, Jason H. Moore

**Affiliations:** Department of Biological Sciences, North Carolina State University, Raleigh, NC 27695 USA; Institute for Quantitative Biomedical Sciences, Geisel School of Medicine, Dartmouth College, Lebanon, NH 03756 USA

## Erratum

Unfortunately, the original version of this article [1] published with an incorrect citation. The article number has now been updated to **6:**124, which is the correct citation for the article. The Publisher apologizes for any inconvenience caused.
